# Disruption of Swell1/VRAC function impairs initial hemodynamics and activates compensatory leukotriene signaling in zebrafish circulation development

**DOI:** 10.3389/fcell.2025.1719544

**Published:** 2025-12-18

**Authors:** Yen-Tzu Tseng, Chia-Teng Chang, Wei-Chun HuangFu, I.-Hsuan Liu

**Affiliations:** 1 Department of Animal Science and Technology, National Taiwan University, Taipei City, Taiwan; 2 Graduate Institute of Cancer Biology and Drug Discovery, College of Medical Science and Technology, Taipei Medical University, Taipei City, Taiwan; 3 Ph.D. Program for Cancer Molecular Biology and Drug Discovery, College of Medical Science and Technology, Taipei Medical University, Taipei City, Taiwan; 4 TMU Research Center of Cancer Translational Medicine, Taipei Medical University, Taipei City, Taiwan; 5 Research Center for Developmental Biology and Regenerative Medicine, National Taiwan University, Taipei City, Taiwan

**Keywords:** 5-lipoxygenase, arachidonic acid metabolism, circulatory system development, LRRC8A, swel1, VRAC, Zebrafish

## Abstract

**Background:**

Volume-regulated anion channels (VRACs) maintain cell-volume homeostasis, and SWELL1 is their essential subunit. Here, we show that VRAC/Swell1 also regulates initial hemodynamics and vascular development in zebrafish.

**Results:**

Stable *swell1a* and *swell1b* mutant zebrafish lines were established. In *SWELL1*- KO HAP1 cells, VRAC currents were rescued by wild-type, but not mutant, zebrafish *swell1a* or *swell1b* cDNA, confirming the alleles' loss-of-function nature. Microangiography and *Tg(fli1a:eGFP)* imaging revealed hypovolemia, reduced flow, and delayed vessel sprouting by 30 hpf, with severity proportional to allele dosage and partial recovery by 72 hpf. Whole-embryo transcriptomics highlighted upregulation of arachidonic-acid metabolism, especially the 5- lipoxygenase (5LO) axis. Pharmacological 5LO inhibition or the receptor *cyslt1r* knockdown aggravated circulatory defects, whereas leukotriene C4 treatment improved hemodynamics, indicating compensatory 5LO signalling. Thus, Swell1-dependent VRAC activity underpins embryonic hemodynamic stability, and 5LO-derived mediators partially buffer its loss.

**Conclusion:**

These findings link ion-channel function to lipid signalling in vascular development and suggest VRAC/Swell1-5LO cross-talk as a therapeutic target for blood-flow disorders.

## 1 Introduction

Volume-regulated anion channels (VRACs) are crucial for maintaining cell volume homeostasis. The molecular identity of VRAC remained elusive until the discovery of LRRC8A as an essential component (Qiu et al., 2014; Voss et al., 2014). Given that LRRC8A-deficient cells exhibit swelling in hypotonic environments, the gene encoding this protein was also termed *SWELL1* (Qiu et al., 2014). Structural studies show that SWELL (LRRC8) family members SWELL1, 3, 4, and 5 share highly conserved sequences at their N-termini, particularly in first transmembrane domain (TM1) and first extracellular loop (EL1), which line the VRAC channel pore (Kefauver et al., 2018; Qiu et al., 2014; Syeda et al., 2016). These SWELL family members form heteromeric channels that regulate the flow of anions, such as chloride, and organic osmolytes, such as taurine, across the cell membrane in response to changes in cell volume (Planells-Cases et al., 2015; Syeda et al., 2016).

Humans with *SWELL1* deficiency exhibit agammaglobulinemia and a lack of circulating B cells (Sawada et al., 2003). In contrast, mice with *Swell1* deficiency are characterized by impaired T cell development and function and various physical abnormalities such as curly hair, hind limb weakness, progressive hydronephrosis, and sterility (Kumar et al., 2014). Later studies indicate that VRAC is widely expressed across multiple cell types and plays critical roles in maintaining cellular homeostasis, particularly involving cell volume changes (Zhang et al., 2017; Xie et al., 2017; Stuhlmann et al., 2018; Luck et al., 2018; Chen et al., 2019; Formaggio et al., 2019). A recent study revealed that endothelial SWELL1 plays a crucial role in mediating endothelial migration and alignment in response to shear-flow stimulation, while endothelial-specific deletion of *Swell1* leads to hypertension in response to chronic angiotensin II infusion and constricted retinal vessels in a type 2 diabetes mellitus model (Alghanem et al., 2021). These results indicate that SWELL1 plays a role in the homeostasis of hemodynamics. However, the role of SWELL1 during early vasculogenesis/angiogenesis and hemodynamics remains unknown.

The circulatory system is the first functional system to develop during early embryogenesis. After the initial stages of vasculogenesis and cardiogenesis, circulation begins. Various studies have indicated that establishing hemodynamics is critical for further developing the circulatory system. For example, obstruction of cardiac inflow leads to malformation of the outflow tract, the maintenance of microvasculature depends on internal blood flow, and shear stress influences hematopoietic differentiation (Hogers et al., 1999; Hove et al., 2003; Adamo et al., 2009; North et al., 2009; Chen et al., 2012). However, hemodynamics begins to function before critical organs that normally regulate blood flow are functional, such as the liver, kidneys, sympathetic nervous system, and even vascular smooth muscle. This raises the intriguing question of how hemodynamics is initiated and regulated during early embryogenesis. Several key genetic factors have been identified as playing roles in early embryonic hemodynamics, such as *tnnt2* for mediating the heartbeat (Sehnert et al., 2002) and components of endothelial adherens junctions, such as *cdh5* and *msna*, which are essential for vascular barrier integrity (Montero-Balaguer et al., 2009; Wang et al., 2010; Bedell et al., 2012). Interestingly, fluid accumulation and the fusion of intracellular vacuoles during the formation and elongation of the vascular network have been described (Kamei et al., 2006; Mostov and Martin-Belmonte, 2006). Still, the critical genetic factors responsible for this process have yet to be identified.

Volume-sensitive organic osmolyte/anion channel (VSOAC) is believed to account for at least half of osmoregulation during cell regulatory volume decrease (RVD) (Garcia-Romeu et al., 1991; Jackson and Strange, 1993). It is reasonable to speculate that VSOAC also participates in the development of the circulatory system, specifically by regulating blood volume and, consequently, blood flow *via* osmoregulation, which is a key mechanism for redistributing water between cellular compartments and maintaining blood plasma homeostasis. Our group and others have identified two zebrafish genes, *swell1a* (ENSDART00000148138) and *swell1b* (ENSDART00000144732), with protein-coding sequences similar to human *SWELL1* (Yamada et al., 2016; Tseng et al., 2020). Knocking down either *swell1a* or *swell1b* using antisense morpholino oligos (MOs) led to impaired brain ventricle inflation (Tseng et al., 2020), which may be partially influenced by the circulatory system (Schier et al., 1996). Additionally, these morphants exhibited gross abnormalities in circulatory system development, including pericardial effusion, cell accumulation in the posterior blood island (PBI), and impaired blood circulation at 32 h post-fertilization (hpf) (Yamada et al., 2016; Tseng et al., 2020). In this study, we characterized the phenotypes associated with the loss of *swell1a* and *swell1b* in zebrafish, explicitly focusing on their effects on circulatory system development, particularly hemodynamics. We also demonstrated that compensatory mechanisms, including cardiac function and the arachidonate 5-lipoxygenase (5LO) pathway, contribute to the gradual recovery of these phenotypes during development, coinciding with the emergence of other functional systems, such as the sympathetic nervous system.

## 2 Materials and methods

### 2.1 Care and use of experimental animals

AB wild-type (WT), *swell1* mutant lines, and transgenic line *Tg*(*fli1a:EGFP*)^y1^ zebrafish were housed at a density of 2-4 fish per liter in an aquatic facility equipped with an automatic recirculation system. The system was maintained at 28.5 °C with a light/dark cycle of 14/10 h, and the fish were fed live adult brine shrimp twice a day (Wei and Liu, 2014). Embryos were collected after spontaneous spawning, allowed to develop in E3 medium, and staged at six hpf at 28.5 °C using morphological criteria (Kimmel et al., 1995). For the Nifedipine experiment, embryos were cultured in an E3 medium supplemented with 10 µM Nifedipine (Sigma-Aldrich) in 0.1% DMSO after 22 hpf. For leukotriene C4 (LTC4) stimulation, embryos were cultured in an E3 medium supplemented with 640 nM LTC4 (Cayman Chemical, Ann Arbor, Michigan, USA) in 0.4% ethanol after 22 hpf. For bulk RNA Seq, 20 embryos at each time point were pooled together as one sample. For all other experiments, each zebrafish embryo was considered an experimental unit. Embryos from multiple clutches were randomly allocated to experimental or control groups to ensure that each treatment group was represented by embryos from multiple parents, thus reducing batch effects. Because zebrafish embryos do not display sexual differentiation at the stages used in this study (up to 72 hpf), sex as a biological variable was not applicable. An untreated control group was collected for each clutch, and if this control group exhibits >85% mortality or abnormality, the entire clutch was excluded from the experiment. All experimental procedures in this study were reviewed and approved by the Institutional Animal Care and Use Committee of National Taiwan University (NTU-104-EL-00085 and NTU-109-EL-00042) and were performed in accordance with the approved guidelines.

### 2.2 Generation of *swell1a* and *swell1b* mutant line

As previously described, the CRISPR/Cas9 system was used to generate *swell1a* and *swell1b* mutant zebrafish (Lee et al., 2020). Briefly, the Cas9-encoding plasmid pCS2-nzCas9n was linearized by NotI digestion and purified using the GenepHlow Gel/PCR Kit (Geneaid Biotech, New Taipei City, TW). One microgram of linearized DNA template was used to synthesize Cas9 mRNA with the mMESSAGE mMACHINE SP6 Transcription Kit (Invitrogen, Waltham, MA, USA). The guide RNA (gRNA) targeting exon one near the transcription start site was generated as previously described (Tseng et al., 2020). Microinjections were performed at the 1-cell stage in embryos with 100 pg of Cas9 mRNA and 10 pg of gRNA mix per injection, as previously described (Chang et al., 2013).

For genotyping mutant fish, adult zebrafish were anesthetized with 0.016% ethyl 3-aminobenzoate methanesulfonate (MS-222, Sigma-Aldrich), and 10% of the caudal fin was collected using surgical scissors. The removed fin tissue was incubated with 30 µL of alkaline lysis buffer (25 mM NaOH, 0.2 mM EDTA, pH 12) at 95 °C for 30 min, followed by the addition of 30 µL of neutralization buffer (40 mM Tris-HCl, pH 5). To validate the genotype, PCR was performed using the neutralized cell extract as the DNA template with *swell1a* or *swell1b* primer pairs (Supplementary Table 1). PCR products were then analyzed by DNA sequencing (Genomics Biotech, New Taipei City, TW). To verify the mutant sequences at the transcript level, total RNA was extracted from the offspring of mutant fish. RT-PCR was performed using *swell1a* or *swell1b* primer pairs (Supplementary Table 1), followed by DNA sequencing. Heterozygous mutant zebrafish were collected and intercrossed to generate homozygous *swell1a* (*twu0421*) and *swell1b* (*twu0422*) mutant fish. To generate *swell1a* and *swell1b* double knockout (dKO) fish, homozygous *twu0421* and *twu0422* mutant lines were crossed to generate double heterozygous (dHetero) mutant fish, followed by intercrossing of dHetero mutant fish.

### 2.3 Micrography and microangiography

For morphometric analysis of brain ventricles in zebrafish embryos, images were documented using a Leica DM2500 microscope (Leica Microsystems, Wetzlar, DE). The area representing the diencephalic/mesencephalic ventricle (DMv) (Fame et al., 2016) was manually delineated and calculated in ImageJ (Schneider et al., 2012), as previously described (Tseng et al., 2020).

To calculate blood cell flow, zebrafish embryos were anesthetized at the desired developmental time points, and videos of blood vessels in the tail (green box area in Figure 3A) were recorded under a microscope (DM2500, Leica). Blood cell flow in the dorsal aorta (DA) at 30 hpf was measured by manually tracking individual cells or by using Tracker software (version 6.1.2). At 48 and 72 hpf, two arterial intersegmental vessels (aISVs) were randomly selected in each embryo, and the average blood cell flow was determined either manually or using the Tracker software.

For microangiography, TRITC-dextran (20 mg/mL) (Sigma-Aldrich) was injected into the sinus venosus of anesthetized zebrafish embryos. Fluorescent images of the embryos were captured at 30, 48, and 72 hpf under a microscope (DM2500, Leica) (Chen et al., 2006; Zhong et al., 2006). Images of the DA and posterior cardinal vein (PCV) around the middle section of the yolk sac extension were cropped, and the widths of the DA and PCV were calculated by dividing the fluorescence signal area by the length of the image.

For mRNA rescue, 100 pg of *swell1a* mRNA was injected into the 1-cell stage in embryos.

### 2.4 Molecular cloning

The total RNA of embryonic zebrafish was obtained as previously described (Tseng et al., 2020). Briefly, mRNA was extracted from *twu0421* and *twu0422* embryos using TRIzol Reagent (Thermo Fisher Scientific, Waltham, MA, USA). Single-stranded cDNA was synthesized from 2 µg of total RNA with an oligo dT15 primer and SuperScript III Reverse Transcriptase in 20 µL reaction (Thermo Fisher Scientific). The resulting cDNA was used for both cloning and quantitative PCR.

To synthesize mRNA for microinjection with a cistronic expression of a green fluorescence reporter, pT7-*swell1a*-IRES2-EGFP and pT7-*swell1b*-IRES2-EGFP plasmids were utilized as previously described (Tseng et al., 2020). The plasmids were linearized using *Afl*II, and the mRNA encoding *swell1a*-IRES-EGFP and *swell1b*-IRES-EGFP was synthesized using the T7 mMESSAGE mMACHINE kit (Thermo Fisher Scientific). The synthesized mRNAs were aliquoted and stored at −80 °C until use. Before each experiment, the mRNA was mixed with 0.5% phenol red.

For mammalian cell expression, the puromycin resistance gene cassette and polyA signal were amplified from pAS4.1W.RFP-C.Ppuro-aON and pT7-IRES2-EGFP, respectively. The DNA fragments were then cloned into pT7-IRES2-EGFP with *EcoR*V and *Bgl*II to create pT7-IRES2-EGFP-Puro. The coding sequences of *swell1a* and *swell1b* with a Flag tag at the N-terminus were amplified and cloned into pT7-IRES2-EGFP-Puro using Gibson Assembly Master Mix (New England Biolabs, Ipswich, MA, USA), resulting in pT7-*swell1a*-IRES2-EGFP-Puro and pT7-*swell1b*-IRES2-EGFP-Puro, respectively. Zebrafish *twu0421* and *twu0422* mutations were introduced into pT7-*swell1a*-IRES2-EGFP-Puro and pT7-*swell1*-IRES2-EGFP-Puro by site-directed mutagenesis following back-to-back PCR method to create pT7-*twu0421*-IRES2-EGFP-Puro and pT7-*twu0422*-IRES2-EGFP-Puro, respectively.

### 2.5 Transfection

The HAP1 and *SWELL1*-knockout HAP1 (HAP1/SWELL1*) cell lines (Horizon Genomics, Vienna, AT) were cultured in Iscove’s Modified Dulbecco’s Medium supplemented with 10% fetal bovine serum in a 5% CO_2_ incubator. Cells were seeded at 3 × 10^5^ cells per 6-cm culture dish for 24 h, then transfected with the desired vector using TransIT-X2 reagent (Mirus Bio, Madison, WI, USA). After 24 h, transfected cells were plated at 3 × 10^4^ cells per 24-well plate on 12-mm coverslips for an additional 24 h. The transfected cells attached to the coverslips were used for electrophysiology analysis.

### 2.6 Electrophysiology

Whole-cell currents were measured using the Axon MultiClamp 700B amplifier (Molecular Devices, San Jose, CA, USA) and Clampfit software (Version 10.7.0.3, Molecular Devices). Cells were immersed in an isotonic solution (296 mOsm, 90 mM NaCl, 2 mM KCl, 1 mM MgCl_2_, 1 mM CaCl_2_, 10 mM HEPES, 100 mM mannitol, pH 7.4) before exposure to a hypotonic solution (196 mOsm, 90 mM NaCl, 2 mM KCl, 1 mM MgCl_2_, 1 mM CaCl_2_, 10 mM HEPES, pH 7.4). Currents were elicited by a ramp protocol from −100 mV to +100 mV over 200 m, and cells were held at 0 mV for 5 s between voltage ramps. The pipette solution contained the following: 40 mM CsCl, 1 mM MgCl_2_, 1.93 mM CaCl_2_, 5 mM EGTA, 10 mM HEPES, 4 mM Na_2_ATP, 0.5 mM Na_3_GTP, 100 mM Cs-gluconate, pH 7.4 (297 mOsm). Currents were normalized to cell volume, and the current/voltage relationship of maximally activated currents was calculated. The osmolality of all solutions was monitored using a Micro-Osmometer Model 3300 (Advanced Instruments, Norwood, MA, USA)

### 2.7 *In situ* hybridization

To observe the spatial expression of blood vessel- and heart development-related genes during embryonic development, whole-mount *in situ* hybridization was performed as previously described (Chang et al., 2013; Thisse and Thisse, 2008). Briefly, linear DNA templates for mRNA detection were generated by PCR and used for *in vitro* transcription with T7 polymerase to synthesize antisense digoxigenin-labeled riboprobes. Zebrafish embryos were dechorionated, fixed with 4% paraformaldehyde in PBS, and permeablized with proteinase K (10 μg/mL, Amresco, Solon, OH, USA. The fixed embryos were pre-hybridized without riboprobes for 3 hours at 65 °C, followed by overnight hybridization with 50 ng of RNA probe at the same temperature. Subsequently, the hybridized embryos were washed, blocked for 3 h at room temperature, and then incubated with Anti-Digoxigenin-AP Fab fragments (diluted 1:5,000 in blocking solution; Roche Applied Science, Mannheim, DE) with agitation overnight at 4 °C. After washing, the hybridization signals were detected using NBT/BCIP solution (Roche Applied Science) and observed and documented using a Leica Z16-APO microscope (Leica Microsystems).

### 2.8 Bulk RNA sequencing

For transcriptome analysis, dKO and WT embryos were harvested at 30, 48, and 72 hpf. Twenty embryos were homogenized in TRIzol Reagent, mixed with 1-Bromo-3-chloropropane (Molecular Research Center, Cincinnati, OH, USA), and then centrifuged at 12,000 × g for 15 min at 4 °C. The aqueous phase was collected and mixed with isopropanol before being centrifuged at 12,000 × g for 10 min at 4 °C. The pellet was then washed with 75% ethanol, briefly air-dried, and dissolved in DEPC-treated water. The purity and quality of the RNA were checked using SimpliNano-Biochrom Spectrophotometers (Biochrom, Holliston, MA, USA). RNA degradation and integrity were measured using the Qsep 100 DNA/RNA Analyzer (BiOptic, New Taipei City, TW).

For library preparation, 1 μg of total RNA per sample was used as input material, and libraries were generated using the KAPA mRNA HyperPrep Kit (KAPA Biosystems, Wilmington, MA, USA), following the manufacturer’s recommendations. Index codes were added to attribute sequences to each sample. To select cDNA fragments approximately 300–400 bp in length, the library fragments were purified using the KAPA Pure Beads system. The library was amplified using KAPA HiFi HotStart ReadyMix and library amplification primers. PCR products were purified using the KAPA Pure Beads system, and library quality was assessed using the Qsep 100 DNA/RNA Analyzer, the Qubit 2.0 Fluorometer (Thermo Fisher Scientific), and the Agilent Bioanalyzer 2100 system (Agilent Technologies, Santa Clara, CA, USA). The library was sequenced on an Illumina NovaSeq6000 platform (Illumina, San Diego, CA, USA), generating 150 bp paired-end reads.

The original data were transformed into raw sequenced reads by CASAVA base calling and stored in FASTQ format. The quality of raw reads was checked using FastQC and MultiQC (Ewels et al., 2016). Raw reads were filtered using Trimmomatic (v 0.38) (Bolger et al., 2014) to discard low-quality reads, trim adaptor sequences, and eliminate poor-quality bases, using the following parameters: LEADING:3, TRAILING:3, SLIDINGWINDOW:4:15, MINLEN:30. Read pairs from each sample were aligned to the reference genome (*D. rerio*, GRCz11) using HISAT2 software (v 2.1.0) (Kim et al., 2015; Sahraeian et al., 2017). Read counts mapped to individual genes were obtained using FeatureCounts (v 2.0.0) (Liao et al., 2014). “Relative Log Expression” normalization (RLE) was performed using DESeq2 (v 1.26.0) (Love et al., 2014; Schurch et al., 2016). Differentially expressed genes (DEGs) analysis of the two conditions was performed in R using DESeq2, based on a Poisson distribution model (Anders et al., 2013; Li et al., 2016; Maza, 2016). P-values were adjusted using the Benjamini and Hochberg approach to control the false discovery rate (FDR). The top 20 upregulated and downregulated DEGs were selected and analyzed using DAVID Bioinformatics Resources (Huang da et al., 2009; Sherman et al., 2022).

### 2.9 Gene knockdown

To knock down *swell1a* and *swell1b*, antisense morpholino oligos (MOs) (Gene-Tools, Philomath, OR, USA) were used as previously described (Tseng et al., 2020). All MOs were dissolved in distilled water to make a 2 mM stock and diluted to the desired concentration with 0.5% phenol red (Sigma-Aldrich) before use.

CRISPR interference (CRISPRi) was also used for gene-specific knockdown (Qi et al., 2013). A crRNA targeting the non-template strand near the transcriptional start sites of the target genes was selected from CHOPCHOP (Labun et al., 2019) (Supplementary Table 1) and commercially synthesized (Integrated DNA Technologies, Coralville, IA, USA). The crRNA was annealed with tracrRNA and mixed with *nzdCas9n* mRNA (a catalytically inactive Cas9 with D10A and H840A mutations and a nuclear localization signal), followed by microinjection at the one-cell stage in embryos as previously described (Chang et al., 2013).

### 2.10 Quantitative reverse transcriptase polymerase chain reaction (qRT-PCR)

For single-strand cDNA synthesis, 2 μg of total RNA was mixed with oligo-dT primers and SuperScript III reverse transcriptase in 20 μL reaction (Invitrogen, Carlsbad, CA, USA) and incubated for 60 min at 50 °C. To quantitatively analyze the expression level of target genes, 4 μL of 10× diluted cDNA was mixed with 5 μL of iQ SYBR Green Supermix (Bio-Rad, Hercules, CA, USA) and 1 μL of primer set mix (Supplementary Table 1). The PCR reaction conditions were as follows: 95 °C for 10 min, followed by 40 cycles of 95 °C for 15 s and 60 °C for 15 s, with a final extension at 72 °C for 30 s. The ΔΔCT values were calculated by first subtracting the CT of the internal control (*actb1*) from the CT of each target gene to obtain ΔCT, and then subtracting the ΔCT of the control group from that of the experimental group. We present -ΔΔCT so that the direction of expression changes is directly conveyed, with positive values indicating upregulation and negative values indicating downregulation.

### 2.11 Statistical analysis

Statistical analyses were performed using GraphPad Prism 9 (GraphPad Software, San Diego, CA, USA) or RStudio (version 2024.4.2.764; RStudio, PBC, Boston, MA, USA) with R (version 4.4.1, 2024-06-14) for scRNA-seq analysis. Quantitative PCR data were statistically evaluated using unpaired *t*-tests, while bulk RNA sequencing data were analyzed using unpaired *t*-tests with false discovery rate (FDR) adjustment for multiple comparisons. The heart rate, vessel length, and vessel diameter were analyzed using unpaired *t*-test or one-way ANOVA followed by Tukey’s *post hoc* test. The blood cell numbers were analyzed using the Mann-Whitney U test or the Kruskal-Wallis test, followed by Dunn’s *post hoc* test. A P-value less than 0.05 was considered statistically significant. For single-cell RNA sequencing (scRNA-seq) re-analysis, datasets were acquired from Zebrahub (Lange et al., 2023; Lange et al., 2024). Tissue-specific enrichment of gene expression at each time point was determined by comparing expression in a given tissue to all other tissues using the Wilcoxon rank-sum test (Pullin and McCarthy, 2024). To correct for multiple comparisons, the false discovery rate (FDR) was calculated using the Benjamini-Hochberg method, and an FDR of less than 0.05 was considered statistically significant. Unless otherwise stated, all data are presented as mean ± standard error of the mean (SEM).

## 3 Results

### 3.1 VRAC/VSOAC are deficient in *twu0421* and *twu0422* fish

**FIGURE 1:**
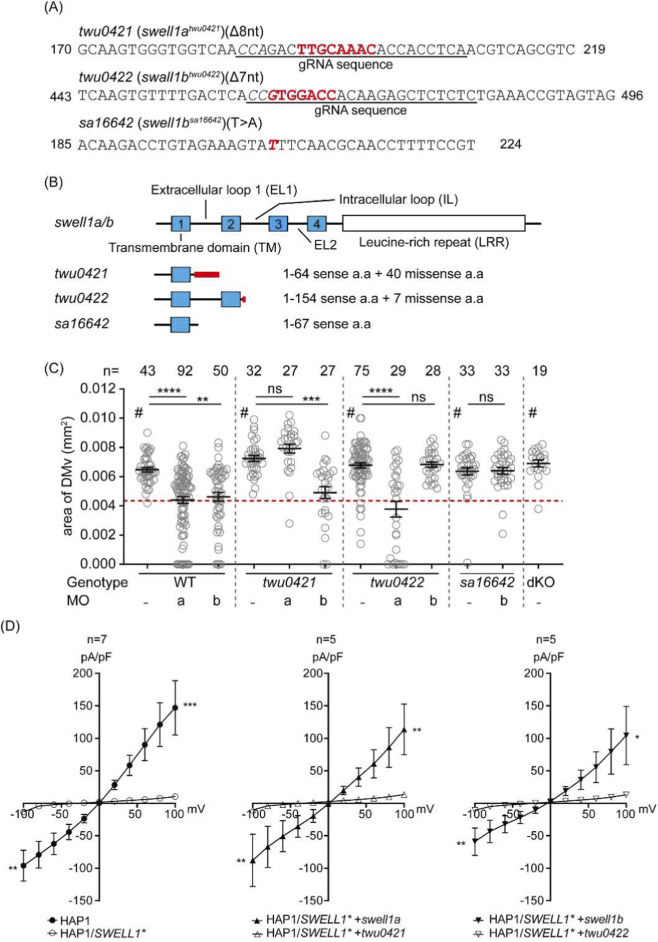
VSOAC deficiency in *swell1a* ^*twu0421/twu0421*^ and *swell1b* ^*twu0422/twu0422*^ mutants. **(A)** Genomic lesions in *swell1a* ^*twu0421*^ (*twu0421*) and *swell1b* ^*twu0422*^ (*twu0422*) were generated using CRISPR/Cas9 with specific guideRNA (gRNA), while the s*well1b* ^*sa16642*^ (*sa16642*) mutant line was obtained from ZIRC. The gRNA-specific sequence is underlined, the protospacer adjacent motif (PAM) is shown in italics, deleted nucleotides in the mutant lines are highlighted in bold red, and a point mutation in *sa16642* is shown in bold italic red. **(B)** Protein domain structure of *swell1a* and *swell1b* and corresponding mutant proteins. The transmembrane domain (TM) is shown as a blue box, and the leucine-rich repeat (LRR) domain is shown as a white box. The location of the missense amino acid mutation in the mutant line is indicated by a thick red line. **(C)** The inflation of the brain ventricle was not impaired in *swell1a* and *swell1b* mutant lines. The diencephalic/mesencephalic ventricle (DMv) area sizes are comparable between WT and *swell1a/b* single mutant, and the double knockout (dKO) line. Morpholinos specifically targeting *swell1a* (*a*MO) and *swell1b* (*b*MO) were used to validate gene knockdown specificity. The mutant lines resist the corresponding MO but remain susceptible to the other. Genotype: WT (wild-type); *twu0421* (*swell1a* ^*twu0421*^); *twu0422* (*swell1b* ^*twu0422*^); *sa16642* (*swell1b* ^*sa16642*^); dKO (*swell1a* ^*twu0421*^; *swell1b* ^*twu0422*^). MO: a (*swll1a* morpholino); b (*swell1b* morpholino). The sample size (n) of each group is indicated at the top of the scatter plot. The data were collected from three biological replicates. ns, no significance; **p < 0.01; ***p < 0.001; ****p < 0.0001; #, no significant difference between each other. **(D)** Whole-cell patch clamp recordings under hypotonic conditions showed current changes in HAP1 cells but not in SWELL1-knockout cells (HAP1/SWELL1*). Overexpression of WT *swell1a* (HAP1/SWELL1* + *swell1a*) or *swell1b* (HAP1/SWELL1* + *swell1b*) restored VSOAC activity. However, overexpression of *twu0421* (HAP1/SWELL1* + *twu0421*) or *twu0422* (HAP1/SWELL1* + *twu0422*) failed to rescue VSOAC activity. The sample size (n) of each group is indicated at the top of the histogram. The data were collected from three biological replicates. *p < 0.05; **p < 0.01; ***p < 0.001.

Using CRISPR/Cas9 genome editing, we generated loss-of-function alleles in *swell1a* and *swell1b*. The *swell1a* mutant allele carries an 8-bp deletion (*c.192_199del*), which introduces a frameshift predicted to generate 40 aberrant amino acids followed by a premature stop codon (*p.C65PfsX104*). The *swell1b* mutant allele carries a 7-bp deletion (*c.462_468del*), resulting in a frameshift predicted to generate 7 aberrant amino acids before a premature stop (*p.W155QfsX161*). Following ZFIN guidelines for line designation, the stable *swell1a* and *swell1b* mutant lines have been named *twu0421* and *twu0422*, respectively. The predicted truncated protein of Twu0421 contains only the first transmembrane (TM1) and part of the first extracellular (EL1) domain. The predicted truncated protein of Twu0422 contains TM1, TM2, and partial intracellular loop (IL) (Figures 1A,B). As comparison, an independent mutant line (*swell1b* ^*+/sa16642*^) harboring a point mutation in *swell1b* (*swell1b* ^*sa16642*^) was obtained from the Zebrafish International Resource Center, and the homozygous mutant (*sa16642*) was generated through breeding. The protein product of the mutated gene *swell1b* ^*sa16642*^ is predicted to be a truncated protein with only the first 67 amino acids at the N-terminus, containing TM1 and part of EL1 (Figures 1A,B).

The brain ventricles of mutant embryos were compared to those of WT embryos to validate the previously observed phenotype (Tseng et al., 2020). In contrast to the previous study, where *swell1* gene knockdown impaired brain ventricle inflation, the mutant embryos’ brain ventricles were comparable to WT embryos (Figure 1C) (Rossi et al., 2015). Furthermore, the mutant lines were insensitive to *swell1* gene knockdown, as *twu0421* and *twu0422* maintained normal brain ventricle inflation after MOs against *swell1a* (*a*MO) and *swell1b* (*b*MO) were introduced, respectively. Interestingly, *a*MO induced an identical small brain ventricle phenotype in *twu0422*, while *b*MO induced the same phenotype in *twu0421* as in the WT. The resistance of the *twu0421* and *twu0422* mutations to their cognate MOs, but not to each other’s MOs, suggests that the disruption of brain ventricle inflation was specifically caused by the loss-of-function induced by MO knockdown. Furthermore, the *twu0421* and *twu0422* mutations might trigger transcriptional adaptation to compensate for the loss-of-function phenotypes (El-Brolosy et al., 2019).

To confirm that the mutant genes are indeed loss-of-function mutations that cannot assemble functional VRAC, WT and mutant *swell1* genes were cloned and overexpressed in SWELL1-knockout HAP1 cells (HAP1/SWELL1*) to perform whole-cell patch clamp experiments. The current change of HAP1/SWELL1* cells under hypotonic conditions was recorded. The current change was significantly reduced in HAP1/SWELL1* cells rescued with *swell1a* ^*twu0421*^ and *swell1b* ^*twu0422*^ compared to those rescued with WT *swell1a* and *swell1b* (Figure 1D). Moreover, the current change in HAP1/SWELL1* cells rescued with *swell1a* ^*twu0421*^ and *swell1b* ^*twu0422*^ was comparable to that in HAP1/SWELL1* cells without rescue (Figure 1D). The inability of *swell1a* ^*twu0421*^ and *swell1b* ^*twu0422*^ to rescue VRAC activity in HAP1/SWELL1* cells, in contrast to WT *swell1a* and *swell1b*, provides functional evidence that these genomic lesions result in loss of function of the respective genes.

### 3.2 Loss of VRAC/VSOAC results in hypovolemia and decreased hemodynamics

**FIGURE 2:**
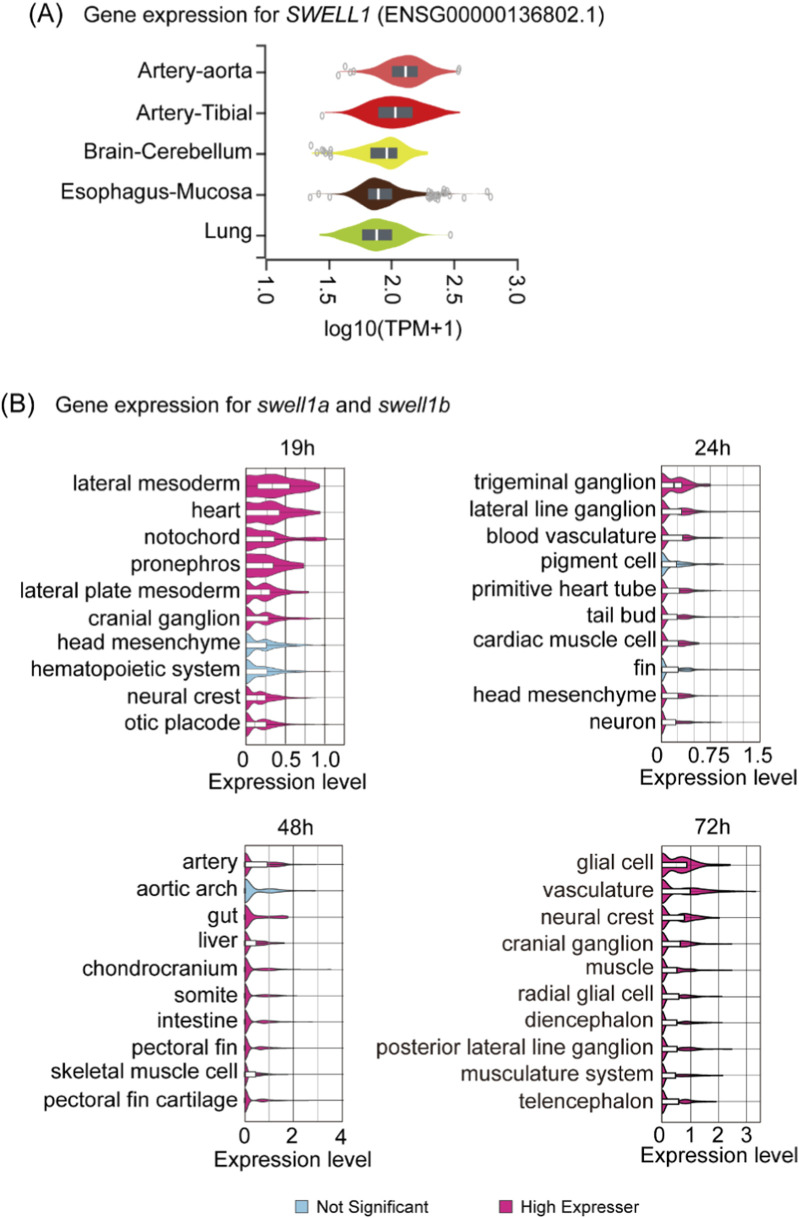
Tissue-specific expression of SWELL1 in humans and its orthologs *swell1a* and *swell1b* in zebrafish embryos. **(A)** Gene expression analysis of *SWELL1* in human tissues based on data from the Genotype-Tissue Expression (GTEx) project. SWELL1 shows the highest expression in arterial tissues, including the aorta and tibial artery, relative to other tissues. **(B)** Spatial and temporal expression profiles of zebrafish *swell1a* and *swell1b* mined from scRNA-seq data. At 19 hpf, *swell1a* and *swell1b* exhibit high expression in cell types related to circulatory system development, including the lateral mesoderm, lateral plate mesoderm, and heart. By 24 hpf, expression remains elevated in blood vasculature and cardiac muscle cells. At 48 hpf, the highest expression is observed in arteries and the aortic arch. By 72 hpf, *swell1a* and *swell1b* expressions are enriched in vasculature and associated cell types. Cell types with significant expression levels are highlighted in pink, while non-significant expression is shown in blue.

VRAC/VSOAC is involved in osmotic regulation and osmolyte displacement, critical mechanisms for modulating fluid balance and morphogenesis during various embryonic events. Consequently, it is reasonable to speculate that Swell1 and VRAC/VSOAC play a role in circulation development. The data from the Genotype-Tissue Expression (GTEx) project (Consortium, 2013) showed that *SWELL1* is expressed highest in the aorta and artery (from tibia) among all measured human tissues (Figure 2A). Similarly, mining the scRNA-seq (Lange et al., 2024) data shows that zebrafish *swell1a* and *swell1b* together exhibit significantly higher expression in cell types related to circulatory system development, including the heart, lateral mesoderm, and lateral plate mesoderm, compared to other embryonic cell types at 19 hpf (Figure 2B). From 24 through 72 hpf, blood vasculature and artery were among the highest expressing cell types across all cell types (Figure 2B). Our previous study reported that *swell1a* and *swell1b* exhibit strong and overlapping expression in the brain ventricle, heart region and sesoryorgans and function redundantly during zebrafish embryogenesis.(Tseng et al., 2020). Whole-mount *in situ* hybridization of *swell1a* also revealed weak expression localized to the region corresponding to the primordial hindbrain channels (Supplementary Figure 1) (Fujita et al., 2011; Isogai et al., 2001).

To further investigate their potential role in the early development of circulation, *twu0421* and *twu0422* were outcrossed to generate *swell1a* ^*+/twu0421*^ and *swell1b* ^*+/twu0422*^ double heterozygous (dHetero) zebrafish, and then *twu0421* and *twu0422* double knockout (dKO) lines were established to study the role of *swell1* and VRAC/VSOAC.

**FIGURE 3:**
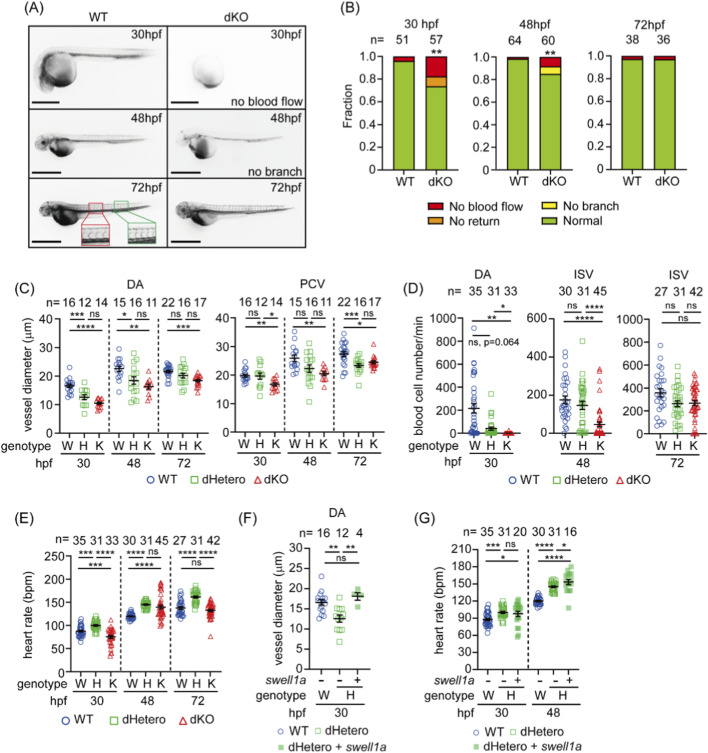
Hypovolemia and reduced hemodynamics in *swell1*-deficient embryos. **(A)** Microangiography revealed impaired circulation in dKO embryos. At 30 hpf, circulation was absent in 17.5% (10/57) of embryos, and in 8.8% (5/57), circulation did not return to the heart. Impaired circulation persisted in 8.3% (5/60) of dKO embryos at 48 hpf, with intersegmental vessels (ISVs) failing to develop in 6.67% (4/60) of embryos (no branching observed). By 72 hpf, impaired circulation was resolved in most dKO embryos. The red box indicates the region used to measure the average diameters of the dorsal aorta (DA) and posterior cardinal vein (PCV), and the green box marks the region used to calculate the number of blood cells flowing through the arterial ISVs. Scale bar: 100 μm. **(B)** Fraction bar diagram showing the circulatory system development in WT and dKO embryos. Sample sizes (*n*) are indicated above the bars. **p < 0.01. **(C)** The vessel diameter was measured from angiographic images. The DA was significantly smaller in dKO embryos at 30, 48, and 72 hpf and in dHetero embryos at 30 and 48 hpf, compared to WT embryos. The PCV diameter was also smaller in dKO embryos at 30, 48, and 72 hpf and in dHetero embryos at 72 hpf. **(D)** The number of blood cells flowing through the DA was significantly decreased in dHetero and dKO embryos at 30 hpf and through the intersegmental vessels (ISVs) in dKO embryos at 48 hpf. **(E)** Heart rate measurements show that dKO embryos have a slower heart rate than WT embryos at 30 hpf but a faster heart rate at 48 hpf. In dHetero embryos, the heart rate is consistently faster than in WT embryos at 30, 48, and 72 hpf. **(F)** Delayed circulation in dHetero embryos was rescued by swell1a mRNA injection. The vessel diameter of the DA in mRNA-injected dHetero embryos was comparable to WT embryos at 30 hpf. **(G)** The heart rate in swell1a mRNA-injected dHetero embryos was significantly faster than in WT embryos at 30 and 48 hpf. The sample size (*n*) for each group is indicated above the scatter plot. The data were collected from three biological replicates for **(C–E)** and from two biological replicates for **(F, G)**. Genotypes: W (wild-type), H (dHetero), K (dKO). ns, no significance; *p < 0.05; **p < 0.01; ***p < 0.001, ****p < 0.0001.

The development of the circulatory system in dKO fish was observed by microangiography, where the red fluorescent dye of TRITC-dextran was pumped from the heart tube into the DA and circulated back to the heart tube *via* the PCV in most WT embryos (49/51, 96%) by 30 hpf (Figure 3A), but was abnormal in about one-fourth of the mutants. Circulation was completely absent in 17.5% (10/57) of the dKO embryos (Figure 3B). In about 8% (5/57) of the embryos, fluorescence was seen in the DA but did not return to the heart (Figure 3B). However, the hearts of these dKO embryos with blood flow defects were still beating. By 48 hpf, the ISVs had developed in WT embryos (Figure 3A). The lack of circulation was still observed in 8.3% (5/60) dKO embryos at 48 hpf. A less severe phenotype was seen in 6.67% (4/60) of the dKO embryos, where circulation developed, but ISVs were absent (Figure 3B). Interestingly, by 72 hpf, the impaired circulation phenotype was no longer observed, and blood circulation was present in most of the dKO embryos (97.2%, 35/36) as well as WT embryos (97.4%, 37/38) (Figures 3A,B). Although the mortality rate was higher in dKO larvae than in WT before 15 dpf, adult dKO fish did not show any circulation-related defects (data not shown).

In line with our speculation that *swell1* and VRAC play roles in circulatory development, we further investigated the phenotype associated with *swell1* deficiency and its impact on vascular developmentby selecting embryos with complete circulation based on microangiography results. The diameters of the dorsal aorta (DA) and posterior cardinal vein (PCV) were measured in angiographic images as indicators of volemia. Starting from 30 hpf, the diameters of the DA and PCV were smaller in dKO embryos, even in those with functional circulation, and this phenotype persisted even with normal circulation at 72 hpf (Figure 3C). On the other hand, the DA diameter remained smaller in dHetero compared to WT at 30 and 48 hpf. In contrast, the diameter of the PCV showed no significant difference between WT and dHetero embryos at these stages. Interestingly, a significantly reduced PCV diameter in dHetero at 72 hpf was observed compared to WT (Figure 3C). These observations indicate a gene dosage effect in the early hypovolemic phenotype.

In addition to hypovolemia, impaired hemodynamics in dKO embryos were also noted. To quantify hemodynamics, we counted the number of blood cells flowing through the DA at 30 hpf and the arterial ISVs (aISVs) at 48 and 72 hpf in a defined period. The number of blood cells flowing through the DA was significantly decreased in dKO embryos compared to WT embryos at 30 hpf (Figure 3D). Notably, the proportion of embryos without blood cell circulation in dKO embryos (96.97%, 32/33) was significantly higher than in dHetero (32.26%, 10/31) and WT embryos (22.85%, 8/35) at 30 hpf. A lower number of blood cells flowing through the aISVs per minute could still be observed in dKO embryos but not in dHetero embryos at 48 hpf (Figure 3D). These observations further support the existence of a gene dosage effect, which might contribute the start time of the blood flow initiation. By 72 hpf, the number of blood cells flowing through the ISVs was comparable among WT, dHetero, and dKO embryos (Figure 3D), indicating recovery of hemodynamics at 72 hpf, which is in line with previous observations from microangiography.

In addition to the volemia, cardiac output is another key player in regulating hemodynamics. The heart rate of dKO embryos is significantly slower than WT at 30 hpf (Figure 3E) when there is no functional sympathetic nervous system to regulate cardiac output. By 48 hpf, when the sympathetic nervous system begins to play its role in regulating cardiac output (Finn et al., 2012), the heart rate in dKO becomes significantly faster than in WT, then comes to a comparable level as WT at 72 hpf (Figure 3E). Interestingly, the heart rate of dHetero embryos is significantly higher than that of WT embryos at 30, 48, and 72 hpf (Figure 3E). Taken together, we observed hypovolemia, bradycardia, impaired circulation, and hemodynamics with the loss of function of *swell1*/VRAC at 30 hpf with a gene dosage effect. In addition, although the circulation phenotype of dKO is restored in angiography and blood cell counts at 72 hpf, the DA diameter and ISV development remain affected, suggesting an unidentified compensatory mechanism restored the hemodynamics to a different homeostasis not identical to the physiological condition in the WT.

To validate that the impaired circulation phenotype was specifically due to the loss of *swell1*/VRAC with a gene dosage effect, an mRNA rescue assay was performed in dHetero. To this end, *swell1a* mRNA was injected into dHetero embryos at the one-cell stage. Accordingly, the diameter of the DA in embryos injected with *swell1a* mRNA was significantly increased to a comparable level as the WT at 30 hpf (Figure 3F). Despite the rescue of the DA width, the heart rate was unaffected by *swell1a* mRNA at 30 hpf but upregulated at 48 hpf to an even higher level (Figure 3G). This result suggests that the hypovolemia at 30 hpf is a primary phenotype caused by the loss of *swell1*/VRAC and, at least partially, can be rescued by overexpression of *swell1a* mRNA.

### 3.3 Cardiac output contributes to hemodynamic recovery but does not underlie early defects in *swell1*-deficient embryos

**FIGURE 4:**
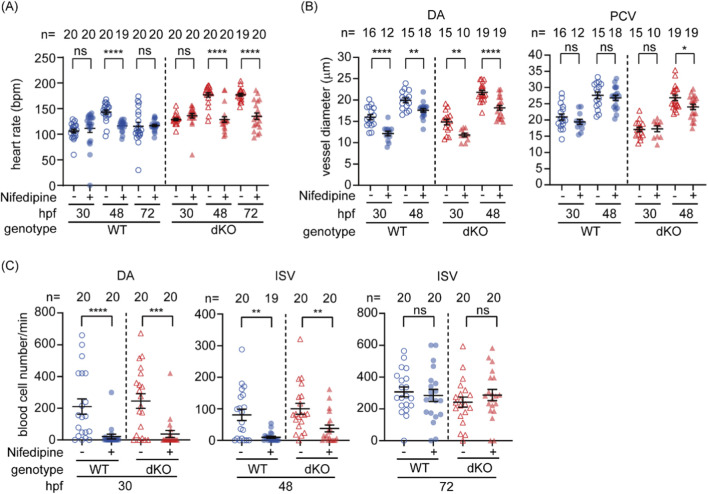
Inhibition of cardiac output by nifedipine further compromises hemodynamics in dKO embryos. **(A)** Nifedipine treatment significantly reduced the heart rate in WT embryos at 48 hpf and in dKO embryos at 48 and 72 hpf. **(B)** The vessel diameter of the dorsal aorta (DA) was significantly reduced in both WT and dKO embryos at 30 and 48 hpf following nifedipine treatment. The posterior cardinal vein (PCV) diameter was also significantly reduced in dKO embryos at 48 hpf. **(C)** Nifedipine treatment significantly reduced blood cell flow (hemodynamics) in both WT and dKO embryos at 30 and 48 hpf, but not at 72 hpf. The sample size (*n*) for each group is indicated above the scatter plot. The data were collected from two biological replicates. Nifedipine: vehicle control with 0.1% DMSO; +, 10 µM nifedipine. ns, no significance; *p < 0.05; **p < 0.01; ***p < 0.001; ****p < 0.0001.

During early development, organs critical in regulating hemodynamics, such as the liver, kidney, and vascular smooth muscle cells, gradually emerge to contribute to hemodynamic regulation after 72 hpf. The recovery of impaired hemodynamics in dKO embryos after 48 hpf coincides with the onset of sympathetic nervous system regulation of heart rate after 44 hpf, suggesting that increased cardiac output may contribute to this compensatory mechanism. To assess the role of cardiac output in the recovery of hemodynamics in the dKO mutant, dKO and WT embryos were immersed in nifedipine, a calcium channel blocker that reduces cardiac output and impairs hematopoiesis in early zebrafish embryos (North et al., 2009). Nifedipine significantly decreased heart rate in both WT and dKO embryos at 48 hpf, eliminating the initially higher heart rate observed in dKO (Figure 4A). DA diameter and blood flow were significantly decreased in both WT and dKO at 30 and 48 hpf (Figures 4B,C). However, by 72 hpf, the blood cell flow in ISVs recovered to comparable level regardless of treatment (Figure 4C). These results suggesting that in addition to the heart rate at 48 hpf there has other emerging regulatory mechanisms by 72 hpf contribute to the gradual recovery of hemodynamics in dKO embryos. Notably, nifedipine produced similar effects on heart rate, DA diameter, and blood cell flow in both WT and dKO embryos at both 30 and 48 hpf (Figures 4A–C), showing that cardiac output responds comparably in both genotypes without selectively exacerbate the perfusion defect in dKO embryos. This supports the conclusion that reduced heart rate or cardiac output alone is insufficient to explain the early circulatory defects caused by *swell1*/VRAC deficiency.

### 3.4 Delayed development is observed in vessels but not blood cells or the heart

**FIGURE 5:**
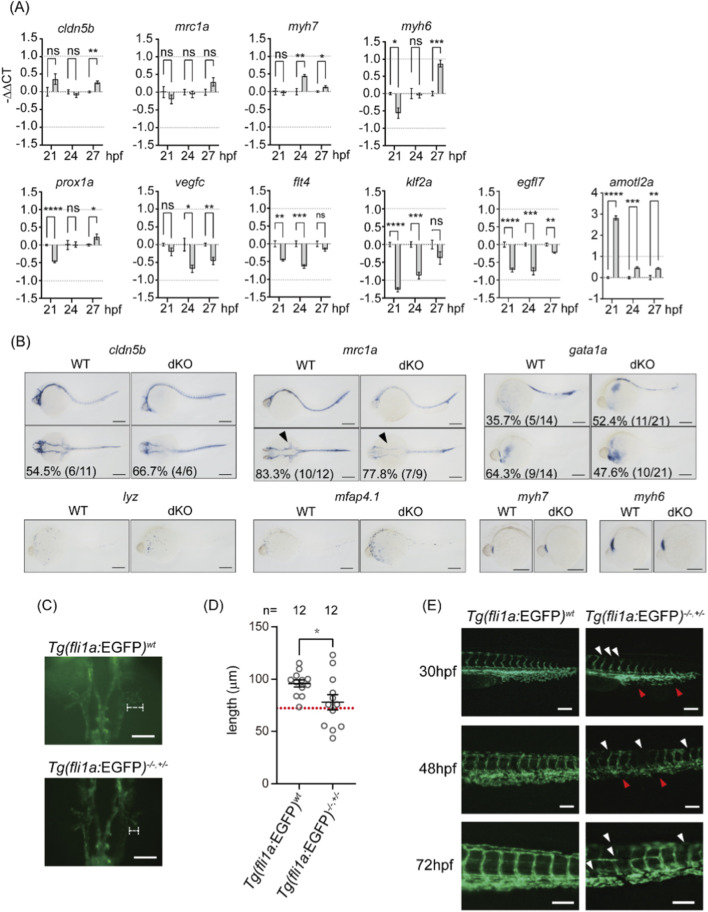
Delayed development is observed in vessels but not blood cells or the heart. **(A)** The expression levels of artery (*cldn5b*), vein (*mrc1a*), heart (*myh7*, *myh6*), lymphatic (*prox1a*, *vegfc*, *flt4*), and endothelial integrity (*klf2a*, *egfl7*, *amotl2a*) markers at 21, 24 and 27 hpf embyros were analyzed by quantitative RT-PCR (qRT-PCR). The gray bars represent the −ΔΔCt values comparing dKO to WT. ns, no significance; *P < 0.05, **p < 0.01, ***p < 0.001, ****p < 0.0001. **(B)** Whole-mount *in situ* hybridization (WISH) analysis at 30 hpf embryo showing the developmental patterns of arteries (*cldn5b*), veins (*mrc1a*), red blood cells (*gata1a*), macrophages (*lyz*), neutrophils (*mfap4.1*), atrium (*myh7*), and ventricle (*myh6*). The lower panel of *cldn5b* and *mrca1* show dorsal views of embryos, while all other images show lateral views. The arrow head indicated the CCV. Scale bar, 200 μm. **(C)** The CCV in WT and dKO embryos at 24 hpf. The length of the extended CCV is outlined by a white dotted line. Scale bar: 100 μm. **(D)** Quantification of CCV length shows significant shortening in Tg(*fli1a*:EGFP); *swell1a* ^*twu0421/twu0421*^; *swell1b* ^*+/twu0422*^ (Tg(*fli1a*:EGFP)^−/−,±^) embryos. The lower normal boundary (72.43 μm) was defined as the mean value minus two standard deviations in WT embryos (indicated by a red dotted line). Shortened CCV was observed in 41.67% (5/12) of Tg(*fli1a*:EGFP)^−/−,±^ embryos. Sample sizes (*n*) are indicated above the scatter plot. The data were collected from two biological replicates. *p < 0.05. **(E)** Delayed development of the dorsal longitudinal anastomotic vessel (DLAV) and ISVs in Tg(*fli1a*:EGFP)^−/−,±^ embryos at 30, 48, and 72 hpf. White arrowheads indicate impaired DLAV and ISV development, while red arrowheads highlight impaired CV development. Scale bar: 100 μm.

To better understand how *swell1* contributes to hemodynamics, we examined a panel of cardiac, vasculature, and blood cell development markers using qPCR and whole-mount *in situ* hybridization. qPCR results indicated that levels of *cldn5b* (arteries) (Xie et al., 2010)and *mrc1a* (veins) (Wu et al., 2014) between WT and dKO embryos were comparable at 21 and 24, and 27 hpf, except *cldn5b* exhibited significant but modest changes (|log2(fold change)| < 1) at 27 hpf (Figure 5A), suggesting that artery and vein differentiation during vasculogenesis is not affected by the loss of *swell1* before the initiation of circulation. *Myh6* (atrium) and *myh7* (ventricles) (Halabi et al., 2022) exhibited significant but modest changes (|log2(fold change)| < 1) (Figure 5A). Additionally, the pattern of *myh6* being downregulated earlier (at 21 hpf) and upregulated later (at 27 hpf), along with *myh7* upregulation at 24 hpf (Figure 5A), indicates that heart differentiation is likely intact. Lymphatic development markers, including *prox1a*, *vegfc*, and *flt4* (Kuchler et al., 2006), showed significant but modest changes (|log2(fold change)| < 1) at various time points (21, 24, and 27 hpf) (Figure 5A), indicating that lymphatic endothelium might be moderately affected by the loss of *swell1*. Furthermore, markers related to endothelial polarity and arrangement, such as *klf2a* and *egfl7* (Dietrich et al., 2014; Parker et al., 2004), are significantly and/or modestly downregulated (0 > log2(fold change) > −1), particularly at 21 and 24 hpf, immediately before the initiation of circulation (Figure 5A). Conversely, *amotl2a*, also involved in endothelial polarity and migration (Wang et al., 2011), is drastically upregulated, especially at the earlier time point of 21 hpf (Figure 5A). The whole-mount *in situ* hybridization showed a noticeable delay in dKO common cardinal vein (CCV) development (*mrc1a*, 7/9, 77.8%) compared to WT (Figure 5B). The expression pattern of blood cells and heart are comparable in dKO and WT embryos (Figure 5B). These data show that the expression levels of cardiac, vascular, and blood cell developmental markers do not exhibit substantial changes between dKO and WT embryos at early developmental stages. This indicates that the hemodynamic defects in dKO embryos are not caused by major abnormalities in heart, vessel, or blood cell development.

We asked whether and how much blood flow defects in *swell1* mutants were contributed from loss of vessel formation. To observe the vessel development defects in *swell1* mutant larvae, the transgenic zebrafish line *Tg*(*fli1a:EGFP*) (Lawson and Weinstein, 2002) with swell1a knockout (*Tg*(*fli1a:EGFP*); *swell1a* ^*twu0421/twu0421*^) was crossed with dKO fish to generate *swell1a* ^*twu0421/twu0421*^; *swell1b* ^*+/twu0422*^ embryos with EGFP signal at vessel endothelial cells (*Tg*(*fli1a*:EGFP);*swell1* ^−/−,+/−^). While the green fluorescent signals appeared in the dorsal aorta and posterior cardinal veins in all observed embryos at 24 hpf, the development of the CCV was delayed (Figure 5C), as the length of the extended CCV was significantly shorter in *Tg*(*fli1a*:EGFP));*swell1* ^−/−,+/−^ embryos compared to *Tg*(*fli1a*:EGFP) embryos (Figure 5D).

To evaluate the penetrance, the mean length of the extended CCV in the WT embryos (95.87 µm) minus two standard deviations (2 × 11.72 µm) was defined as significantly shorter (∼72.43 µm) and phenotypically abnormal. In total, 41.67% (5/12) of *Tg*(*fli1a*:EGFP);*swell1* ^−/−,+/−^ embryos exhibited CCV defect. This finding aligns with the observed loss of *mrc1a* signal in dKO as detected by whole-mount *in situ* hybridization (Figure 5B). Additionally, the development of the dorsal longitudinal anastomotic vessel (DLAV), intersegmental vessels (ISVs), and cardinal veins (CVs) were delayed in *Tg*(*fli1a*:EGFP);*swell1* ^−/−,+/−^ embryos, not only at 30 hpf but also at 48 and 72 hpf (Figure 5E). These results suggest that early vessel development is impaired in VRAC/VSOAC-deficient embryos, likely due to abnormal blood flow.

### 3.5 The upregulated 5LO pathway ameliorates the initial hypovolemia/hemodynamic phenotype

To dissect the mechanisms involved in phenotype amelioration in dKO mutants, we analyzed the transcriptomes of dKO and WT embryos at 30, 48, and 72 hpf using bulk RNA sequencing.

The expression level of lymphatic (*prox1a*, *vegfc*, *flt4*), blood cell (*gata1a*, *mfap4.1*, *lyz*), heart (*myh6* and *myh7*), and vasculature (*kdr*, *kdrl*, *egfl7*, *scl/tal1*, *amotl2a*, *klf2a*) markers were analyzed in our bulk RNA sequence data. By 30, 48 and 72 hpf, the expression levels were comparable between WT and dKO embryos (Supplementary Figure 2). The only downregulated marker was the vein endothelial marker *mrc1a* at 30hpf (Supplementary Figure 2). This finding corresponds with previous results from whole mount *in-situ* hybridization.

**FIGURE 6:**
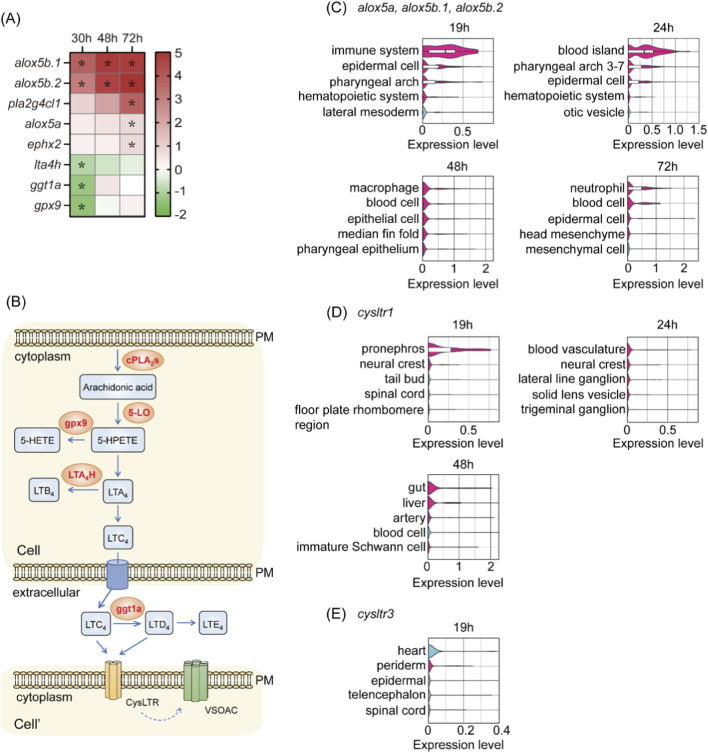
The arachidonic acid metabolism pathway was significantly altered in dKO embryos. **(A)** Heatmap of differentially expressed genes (DEGs) involved in the arachidonic acid metabolism pathway at 30, 48, and 72 hpf in dKO embryos compared to WT embryos. Upregulated genes (*alox5b.1*, *alox5b.2*, *pla2g4c.1*, *alox5a*) and downregulated genes (*lta4h*, *ggt1a*, *gpx9*) are shown. *p < 0.05. **(B)** Arachidonic acid is released by cytoplasmic phospholipase A2 (cPLA2s) and converted to 5-hydroperoxyeicosatetraenoic acid (5-HPETE) by 5LO. The pathway leads to the production of cysteinyl leukotriene (LTC4, LTD4, and LTE4), which signal *via* cysteinyl leukotriene receptors (CysLTRs). The cell region is highlighted with a light orange box. PM, plasma membrane. Cell and Cell’ denote two different cells. **(C–E)** Single-cell RNA-seq re-analysis shows the spatial and temporal expressions of 5LO (*alox5a*, *alox5b.1*, and *alox5b.2*) **(C)**, *cysltr1* **(D)**, and *cysltr3* **(E)**. **(C)** At 19 and 24 hpf, the expression of 5LO homologs was enriched in hematopoietic progenitors, including blood islands and immune cells. At 48 and 72 hpf, expression remained prominent in immune cells and blood cells. **(D)** *cysltr1* expression was highly enriched in the vasculature, including arteries, at 24 and 48 hpf. **(E)** *cysltr3* showed its highest expression in the heart at 19 hpf but was not particularly enriched in the circulatory system.

The top 20 upregulated and downregulated differentially expressed genes (DEGs) at each time point were analyzed using DAVID Bioinformatics Resources (Sherman et al., 2022). Functional annotation revealed that the arachidonic acid (AA) metabolism pathway was significantly enriched (P = 0.035) (Figure 6A; Meirer et al., 2014). The most drastically altered genes included the upregulated *alox5b.1* and *alox5b.2* and the downregulated *lta4h*, *ggt1a*, and *gpx9*. The upregulated genes encode 5LO (*alox5a*, *alox5b.1*, and *alox5b.2*), which converts AA into 5-hydroperoxyeicosatetraenoic acid (5-HPETE), leading to the biosynthesis of leukotrienes (Radmark and Samuelsson, 2009). Meanwhile, the downregulated genes encode enzymes that divert the pathway away from the biosynthesis and accumulation of leukotriene C4 (LTC4) (Figure 6B), suggesting that LTC4 might play a role in the development of early hemodynamics and partially compensate for the loss of *swell1*/VRAC. The upregulation of 5-LO genes was validated by qPCR (Supplementary Figure 3). Expression of *alox5a* was significantly increased in dKO embryos at 30 and 48 hpf, and showed an elevated but non-significant trend at 72 hpf. The *alox5b.1* expression was also higher in dKO embryos, with statistical significance observed only at 30 hpf. For *alox5b.2*, a significant increase was detected only at 30 hpf. The discrepancies between the qPCR and RNA-seq expression patterns for *alox5b.1* and *alox5b.2* may reflect their overall lower expression levels compared with *alox5a*. Together, these results support the DEG analysis showing that 5-LO genes are upregulated in dKO embryos.

Single-cell RNA-seq data mining indicates that the enzymes involved in the 5LO pathway, specifically *alox5a*, *alox5b.1*, and *alox5b.2*, are highly enriched in hematopoietic cells and their progenitors, including the blood island but not in vasculatures and endothelial cells, after 19 hpf (Figure 6C). This suggests a significant role for leukotriene synthesis in these regions during critical hematopoiesis and vascular development stages. LTC4 binds to cysteinyl leukotriene receptors (CysLTRs) at the cell membrane to exert its signal. In the zebrafish genome, only *cysltr1* and *cysltr3* are officially annotated in the current Ensembl genome browser (release 112, GRCz11). Interestingly, the expression of *cysltr1* is highly enriched in blood vasculature and arteries among all cell types at 24 and 48 hpf (Figure 6D). In contrast, *cysltr3,* although not particularly enriched in the circulatory system, was expressed highest in the heart (Figure 6E; Supplementary Figure 4B). These observations suggest that the 5LO pathway *via Cysltr1* plays a crucial role in early hemodynamics and compensates for the loss of *swell1*/VRAC.

**FIGURE 7:**
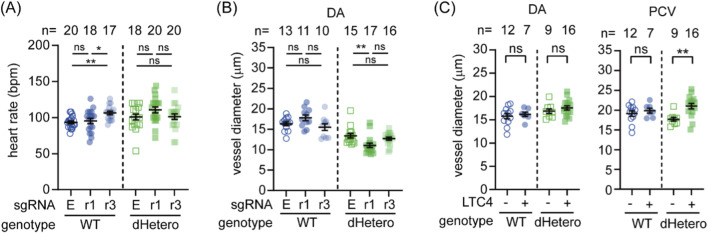
LTC4 participate in the genetic compensation for the loss of *swell1* and ameliorate the hemodynamic phenotype. **(A,B)** Effects of *cysltr1* and *cysltr3* knockdown on early embryonic hemodynamics were evaluated at 30 hpf. **(A)** Heart rate in WT and dHetero embryos following CRISPR interference targeting *cysltr1* (r1) and *cysltr3* (r3). Knockdown of *cysltr3* significantly reduced heart rate in WT embryos, while no significant effect was observed in dHetero embryos. **(B)** Vessel diameter of the dorsal aorta (DA) remained unaffected in WT embryos after *cysltr1* or *cysltr3* knockdown. However, *cysltr1* knockdown further reduced DA diameter in dHetero embryos. **(C)** Rescue effects of LTC4 treatment on early hemodynamic defects at 30 hpf. Vessel diameters were unaffected in WT, but the diameter of the posterior cardinal vein was significantly increased in dHetero embryos after LTC4 treatment. The sample size (*n*) for each group is indicated above the scatter plot. The data were collected from two biological replicates. LTC4: vehicle control with 0.4% ethanol; +, 640 nM nifedipine. ns, no significance; *p < 0.05; **p < 0.01.

To validate the role of AA and 5LO in early embryonic hemodynamics, we knocked down *cysltr1* and *cysltr3* in WT and dHetero embryos using CRISPR interference (Qi et al., 2013). The knockdown efficiency was validated by quantitative RT-PCR (Supplementary Figure 5). While neither knockdown affected the DA diameter in WT embryos, the knockdown of *cysltr3* influenced the heart rate (Figures 7A,B). Conversely, the knockdown of *cysltr1* further reduced the DA diameter in dHetero embryos (Figure 7B) without affecting heart rate (Figure 7A) or blood cell flow (data not shown; P-value of Kruskal-Wallis test: WT, 0.7642; dHetero, 0.276). These results also align with previous single-cell RNA-seq data mining results (Figures 6D,E), which showed that *cysltr1* is enriched in blood vasculature at 24 hpf and *cysltr3* is highest expressed in the heart at 19 hpf. In complementary gain-of-function experiments, immersion of embryos in LTC4 from 22 hpf did not significantly change DA diameter at 30 hpf in either genotype (Figure 7C), but significantly increased PCV diameter in dHetero embryos, with no effect in WT (Figure 7C). These results underscore the significant role of the 5LO pathway in response to the loss of *swell1* and in modulating volemia and hemodynamics during embryogenesis.

## 4 Discussion

In this study, we investigated the role of Swell1 in circulatory system development using zebrafish models harboring targeted mutations in *swell1a* and *swell1b*. Our findings demonstrate that losing Swell1/VRAC function leads to impaired circulatory system development, characterized by hypovolemia, decreased hemodynamics, and delayed vessel formation. Furthermore, we observed that compensatory mechanisms involving the 5LO pathway ameliorate these phenotypes in dHetero mutants.

Our observations indicate that the impaired hemodynamics in Swell1-deficient embryos occur at developmental stages before the functional maturation of other regulatory systems such as the sympathetic nervous system, liver, and kidneys. The sympathetic nervous system begins to regulate the circulatory system only after 44 hpf (Finn et al., 2012). Pericytes first associate with arterial and intersegmental vessels around 48 hpf, stabilizing endothelial tubes but not yet contributing contractile force (Ando et al., 2016; Stratman et al., 2017). Between 48 and 72 hpf, these mural precursors begin differentiating into vascular smooth-muscle cells (vSMCs), which progressively acquire circumferential alignment and contractile features as arterial flow and caliber increase from 2–4 dpf (Ando et al., 2019; Stratman et al., 2020). By 3–5 dpf, maturing vSMCs begin to provide functional vessel tone, marking the developmental onset of mural cell–mediated vascular contraction (Ando et al., 2021). Similarly, the kidneys and liver, which are crucial for maintaining ionic homeostasis and oncotic pressure, respectively, become vascularized and functional after 48 hpf (Outtandy et al., 2019) and 55 hpf (Korzh et al., 2008; Lorent et al., 2010; Yin et al., 2012), well after the onset of circulation. Additionally, the expression of angiotensinogen in the liver, a key component of the renin-angiotensin system involved in blood pressure regulation, begins only after 72 hpf (Cheng et al., 2006). This temporal gap suggests that Swell1/VRAC channels are pivotal in hemodynamic initiation when other regulatory mechanisms are not yet operational. The recovery of hemodynamics in dKO embryos after 48 hpf coincides with the gradual maturation of these systems, highlighting the significance of Swell1 in the initial stages of circulatory development.

With the emergence of other cells and organs that play roles in modulating hemodynamics, the delayed development of the CCV and most of the impaired measurements in dKO embryos reach levels comparable to WT embryos. However, we still observed defective development of the DLAV and ISVs in mutant embryos, even at 72 hpf (Figure 5). As hemodynamics plays a critical role in the continued growth of these vessels (Goetz et al., 2014; Yin et al., 2024), our observations suggest that Swell1 continues to play a role in regulating hemodynamics even at 72 hpf. Interestingly, the phenotypes observed in our study indicate a gene dosage effect, where dHetero embryos exhibited intermediate phenotypes between WT and dKO embryos. This was evident in parameters such as DA diameter, heart rate, and blood cell flow (Figure 3). The partial loss of Swell1 function in dHetero indicates that a threshold level of Swell1/VRAC activity is necessary for normal circulatory development and hemodynamics.

At 30 hpf, impaired perfusion can in principle reflect altered cardiac pump performance, defect vascular properties (e.g., lumen integrity or tone), or a reduced in effective circulating blood volume. Functionally, dKO embryos echibit a markedly decreased blood-cell flux along with reduced DA and PCV calibers, indicating diminished hemodynamic throughput and arguing against a vasodilatory or low-resistance state (Figure 3). Importantly, microangiography revealed reduced vascular filling without detectable extravascular dye leakage, suggesting that the defect is not attributable by gross compromise of vascular barrier integrity. In parallel, we found no evidence for delayed cardiac development (Figure 5; Supplementary Figure 2) and no significant reduction in cardiac output–related measures (Figure 4). Blood-lineage markers were not decreased, and in some cases elevated, arguing against impaired hematopoiesis, and endothelial polarity and lumen-formation markers were comparable or increased, indicating that primary defects in lumenogenesis are unlikely (Figure 5; Supplementary Figure 2). Taken together, although subtle timing effects cannot be completely excluded, the integrated functional and molecular evidence strongly supports reduced effective circulating blood volume, functional hypovolemia, as the most parsimonious explanation for the early perfusion defect in dKO embryos.

Similar to the circulatory system, a classical study demonstrated that the initial inflation of the brain ventricle during early embryogenesis depends on barrier integrity and osmolarity gradients (Lowery and Sive, 2005). Our previous study showed that *swell1*/VRAC knockdown impaired brain ventricle inflation at 24 hpf when hemodynamics is not yet involved in brain ventricle morphogenesis (Tseng et al., 2020; Lowery and Sive, 2005). However, the brain ventricle was normally formed in the *twu0421*, *twu0422*, *sa16642*, and dKO mutant embryos (Figure 1). The phenotypic mismatch between knockdown and knockout embryos has been systematically demonstrated (Rossi et al., 2015), and nonsense-mediated mRNA decay (NMD)-mediated transcriptional adaptation has been suggested to play a role in this phenomenon (El-Brolosy et al., 2019; Ma et al., 2019). Furthermore, transcriptome profile shifts can be transgenerational *via* global chromatin signature modification (Jiang et al., 2022). Since at least part of the transcriptome profile changes functionally compensate for the lesioned genome, this phenomenon opens a window to probe for unknown genetic players during embryogenesis. Following this principle, in this study, the 5LO pathway was found to play a role in the disrupted embryonic hemodynamics due to the loss of Swell1/VRAC.

A recent study indicated that the absence of endothelial Swell1 exacerbates hypertension and decreased retinal blood perfusion induced by chronic infusion of angiotensin II or in a dietary-induced type 2 diabetes mellitus model (Alghanem et al., 2021). Interestingly, significantly upregulated DEGs due to the loss of endothelial Swell1 include *Alox5ap*, the co-enzyme of 5LO that assists in converting AA into 5-HPETE. In line with this finding, our study shows that the expression of *swell1* is highly enriched in endothelial/vascular cells, especially in arteries and their progenitor cells after 19 hpf but not at 16 hpf (Figure 2). This suggests that the lumen formation of the DA and PCV might be affected by the loss of *swell1*, but not the early migration and convergence of arterial and venous endothelial cells towards the notochord to form the DA and PCV. In the context of this study, it is intriguing to consider what triggers induce the activation of VRAC in the endothelium, thereby contributing to initial volemia and consequently hemodynamics.

VRACs have been shown to play a critical role in helping cells cope with volume changes by mediating RVD through the efflux of anions and organic molecules, such as taurine and glutamate. Regulatory cell volume changes occur during various cellular activities, including proliferation, apoptosis, hypertrophy, and drastic changes in cell shape. Indeed, evidence shows that the inhibition of Swell1/VRAC, either pharmacologically or genetically, perturbs cell proliferation (Xue et al., 2018), apoptosis (Maeno et al., 2000), and hypertrophy (Chen et al., 2022; Huo et al., 2021; Zhang et al., 2017). This suggests that VRAC plays a critical role in various aspects of cell physiology and may also be influenced by other signals.

While VRACs were originally discovered and studied by immersing cells in hypotonic solutions to induce swelling, it is now recognized that the physiological activation of VRAC is neither driven by drastic hypotonicity nor by direct sensing of membrane stretching. After the molecular identity of Swell1 was discovered, a thorough study using a reconstituted lipid bilayer system indicated that low ionic strength, rather than membrane tension or cytoskeletal disruption, directly triggers Swell1/VRAC activity (Syeda et al., 2016). Additionally, multiple lines of evidence showed that ATP can trigger VRAC activation. For example, ATP efflux blockers reduce VRAC activity (Diaz et al., 1993; Lewis et al., 1993). In addition, applying ATP elicits VRAC activation,(Akita et al., 2011), which can be abolished by Swell1 knockdown (Hyzinski-Garcia et al., 2014). A recent study demonstrated that ATP triggers the activation of P2X receptors, which modulates the phosphorylation of Ser174 on Swell1, thereby enhancing VRAC activation (Wang et al., 2024).

Vascular endothelial growth factor (VEGF) signaling through its receptor VEGFR2 is fundamental to vasculogenesis and angiogenesis. VEGF signaling leads to the phosphorylation of Akt2 and ERK. It activates NADPH oxidases (NOXs), producing reactive oxygen species (ROS), which serve as signaling molecules in endothelial function and vascular remodeling (Bekhite et al., 2016; Schmelter et al., 2006; Ushio-Fukai and Alexander, 2004). Recent studies suggest that Swell1/VRAC may interact with NOX enzymes, influencing ROS production and downstream signaling pathways. Swell1 has been shown to have direct interactions with various membrane-associated NOXs, including NOX1, NOX2, NOX4, and p22phox (Choi et al., 2016; Choi et al., 2021; Huo et al., 2021). Moreover, the activation of Swell1/VRAC can be modulated by low pH requiring NOX-derived H_2_O_2_ (Wang et al., 2017), indicating that Swell1/VRAC activity is intertwined with NOX activity and ROS production. Given that circulatory system development relies on NOX-eNOS-ROS signaling (Lohela et al., 2009; Olsson et al., 2006), it is plausible that Swell1/VRAC contributes to volemia and hemodynamics by modulating NOX activity. Furthermore, evidence demonstrates that Swell1 influences Akt2 phosphorylation (Alghanem et al., 2021; Zhang et al., 2017), linking it directly to the VEGF-VEGFR2-Akt2 signaling axis. This suggests that Swell1/VRAC may play a role in vascular development by modulating key signaling pathways downstream of VEGF. Beyond biochemical signaling, a previous study demonstrated that TRPM7, a mechanosensitive ion channel, is involved in endothelial cell adhesion, migration, and tube formation *via* modulation of myosin light chain phosphorylation and ERK signaling (Zeng et al., 2015). The finding that TRPM7 can activate VRAC through its physical interaction with SWELL1 (Numata et al., 2021) adds another potential layer of regulation for the role of Swell1/VRAC in hemodynamics and volemia.

Leukotrienes have been considered key contributors to hypertension, probably due to their direct regulatory role in the circulatory system or indirectly *via* the renin-angiotensin system (Smedegard et al., 1982; Stanke-Labesque et al., 2001; Stanke-Labesque et al., 2002; Stenmark et al., 1983). While CysLTR2 is generally considered to have a more prominent role in the cardiovascular system due to its high expression in the heart (Heise et al., 2000; Takasaki et al., 2000), it is increasingly recognized that both CysLTR1 and CysLTR2 play important but distinct roles in cardiovascular diseases (Colazzo et al., 2017). Studies performed on human umbilical vein endothelial cells (HUVECs) indicate that signaling through CysLTR2 compromises the integrity of the cell barrier due to elevated cell contraction, whereas signaling through CysLTR1 modulates HUVEC proliferation involving ERK phosphorylation (Duah et al., 2013). Interestingly, a previous study indicated that CysLTR1 antagonists can block VRAC activity (Holm et al., 2013), and this effect can be independent of the presence of *Cysltr1* (Figueroa et al., 2019), suggesting the possibility that leukotrienes can directly modulate VRAC activity.

In the context of our study, the compensatory upregulation of the 5LO pathway in *swell1*-deficient embryos may enhance the production of cysteinyl leukotrienes, particularly LTC4, and signaling through Cysltr1 to help restore hemodynamic stability. This is supported by a study in adult mice, which found that loss of endothelial Swell1/VRAC leads to exacerbated hypertension and retinal hypoperfusion due to vessel constriction (Alghanem et al., 2021). Interestingly, the 5LO pathway was upregulated in both their and our studies, further supporting the notion that both Swell1/VRAC and leukotrienes play a role in the homeostasis of hemodynamics. The fact that inhibition of the 5LO pathway by *cysltr1* knockdown exacerbated the phenotypes, while immersion in LTC4 significantly increased volemia in the PCV of mutant embryos, supports the significance of leukotriene-mediated compensation (Figure 7). Interestingly, the 5LO pathway is significantly enriched in blood cells and their progenitors, while *swell1* and *cysltr1* are enriched in the vasculature (Figures 2, 6). These lines of evidence suggest that blood islands and blood cells signal through cysteinyl leukotrienes to modulate Swell1/VRAC activity in vascular endothelial cells, thereby contributing to the early development and regulation of hemodynamics.

While zebrafish serve as a powerful model organism for studying vertebrate development due to their advantages in manipulating and observing early embryonic stages, inherent differences compared to mammals may limit the direct translation of our findings. Technical limitations, such as the inability to measure osmotic and blood pressures directly or quantify volemia precisely in tiny embryos, may affect the interpretation of some results. To more precisely define the tissue-specific roles of Swell1/VRAC, future studies employing conditional knockout approaches will be essential.

Given the findings in adult mice showing that loss of endothelial Swell1/VRAC leads to exacerbated hypertension and retinal hypoperfusion due to vessel constriction (Alghanem et al., 2021), our study in zebrafish embryos underscores the importance of Swell1/VRAC function across developmental stages. The upregulation of the 5LO pathway observed in both mice and zebrafish studies suggests a conserved compensatory mechanism involving leukotriene signaling in response to Swell1 deficiency. Future investigations should focus on elucidating the molecular mechanisms underlying Swell1/VRAC function in adult physiology and pathophysiology, particularly in the context of hypertension and vascular diseases. Exploring how leukotrienes modulate VRAC activity and affect vascular function in adult models may provide deeper insights into their roles in vascular biology. Additionally, studying the interaction between VRAC function and leukotriene signaling at the cellular and molecular levels might reveal novel therapeutic targets towards hypertension and other cardiovascular diseases.

## 5 Conclusion

Our study demonstrates that Swell1/VRAC plays a role in circulatory system development by regulating volemia and hemodynamics during embryogenesis. The loss of *swell1* leads to impaired hemodynamics and vessel formation, likely due to hypovolemia. Compensatory mechanisms involving the 5LO pathway, as well as the development of other regulatory organs and cell types, contribute to phenotypic recovery. These findings advance our understanding of the interplay between ion channel biology and lipid mediator signaling in vascular development and may inform future research on vascular diseases and therapeutic interventions.

## Data Availability

The raw data supporting the conclusions of this article will be made available by the authors, without undue reservation.
