# Correction: Disruption of Swell1/VRAC function impairs initial hemodynamics and activates compensatory leukotriene signaling in zebrafish circulation development

**DOI:** 10.3389/fcell.2026.1782930

**Published:** 2026-01-21

**Authors:** Frontiers Production Office

**Affiliations:** Frontiers Media SA, Lausanne, Switzerland

**Keywords:** 5-lipoxygenase, arachidonic acid metabolism, circulatory system development, LRRC8A, swel1, VRAC, Zebrafish

The funder National Science and Technology Council, Taiwan (114-2313-13-002-047-) was erroneously omitted.

The original version of this article has been updated.

