# Supplementary material for: Disruption of Swell1/VRAC function impairs initial hemodynamics and activates compensatory leukotriene signaling in zebrafish circulation development
Source: Front Cell Dev Biol. 2025 Dec 18;13:1719544. doi: 10.3389/fcell.2025.1719544 (PMC12756422; doi:10.3389/fcell.2025.1719544)
Supplement: Supplementary file 1 [file DataSheet1.pdf]

Supplementary figure

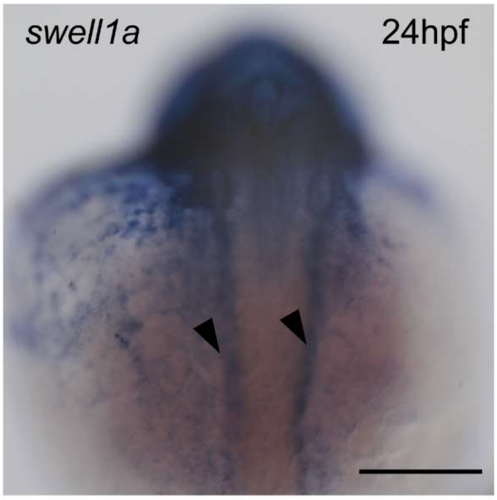

Figure S1. Whole-mount *in situ* hybridization of *swell1a* at 24 hpf. *Swell1a* expression is localized along the region corresponding to the primordial hindbrain channels (arrow head). Dorsal views of embryos are shown. Scale bar: 200 μm.

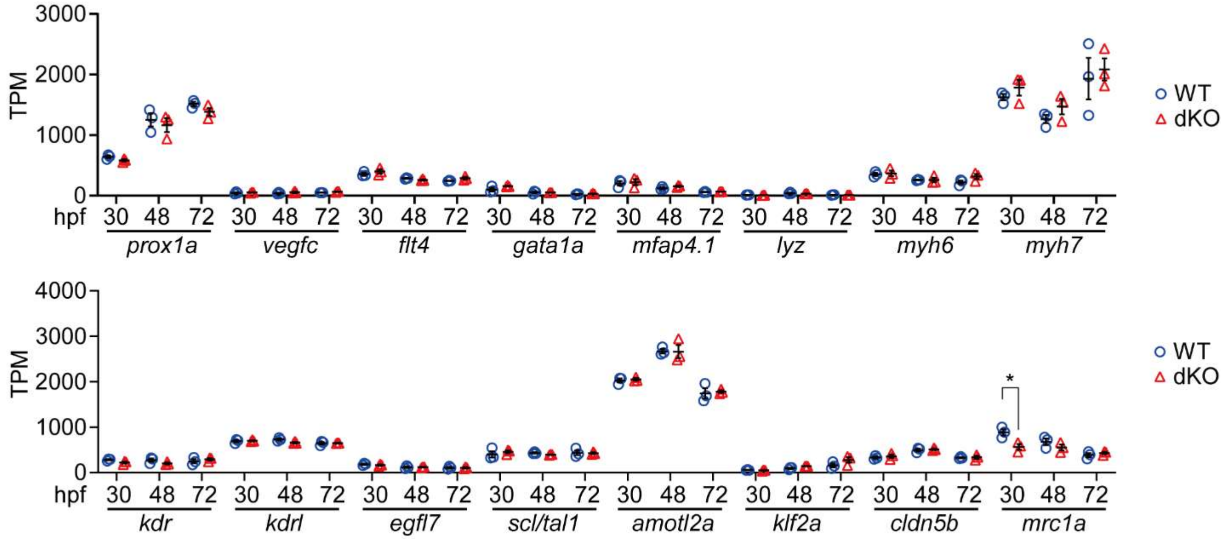

Figure S2. The expression levels of lymphatic (*prox1a*, *vegfc*, *flt4*), blood cell (*gata1a*, *mfap4.1*, *lyz*), heart (*myh6* and *myh7*), vasculature (*kdr*, *kdrl*, *egfl7*, *scl/tal1*, *amotl2a*, *klf2a*), artery (*cldn5b*) and vein (*mrc1a*) markers at 30, 48 and 72 hpf were analyzed by bulk RNA-seq. Each data point has three independent clutches. Adjusted P-values are indicated, with statistical significance denoted as $P < 0.05$.

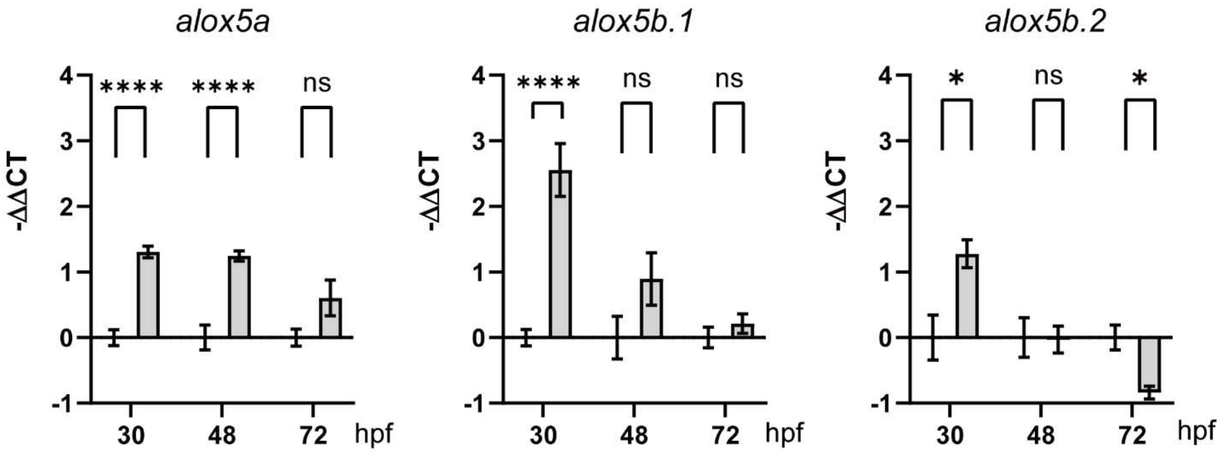

Figure S3. The expression levels of *alo5a*, *alox5b.1* and *alox5b.2* at 30, 48 and 72 hpf embryos were analyzed by quantitative RT-PCR (qRT-PCR). The gray bars represent the −ΔΔCt values comparing dKO to WT. The y-axis -ΔΔCT is mathematically equivalent to log2(fold change). ns, no significance; *P < 0.05, ****P < 0.0001.

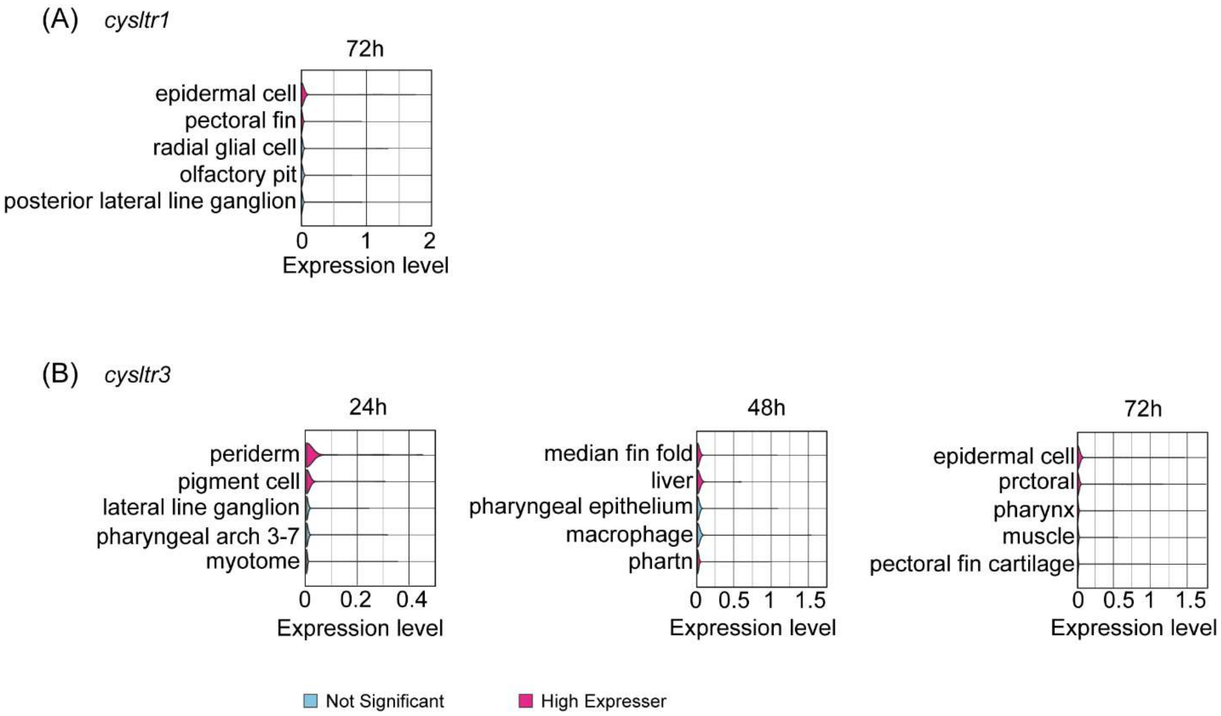

Figure S4. Single-cell RNA-seq re-analysis shows the spatial and temporal expressions of *cysltr1* (A) and *cysltr3* (B). (A) *cysltr1* expression was enriched in the epidermal cell and pectoral fin at 72 hpf. (B) *cysltr3* expression was enriched in the periderm and epidermal cell at 24-72 hpf.

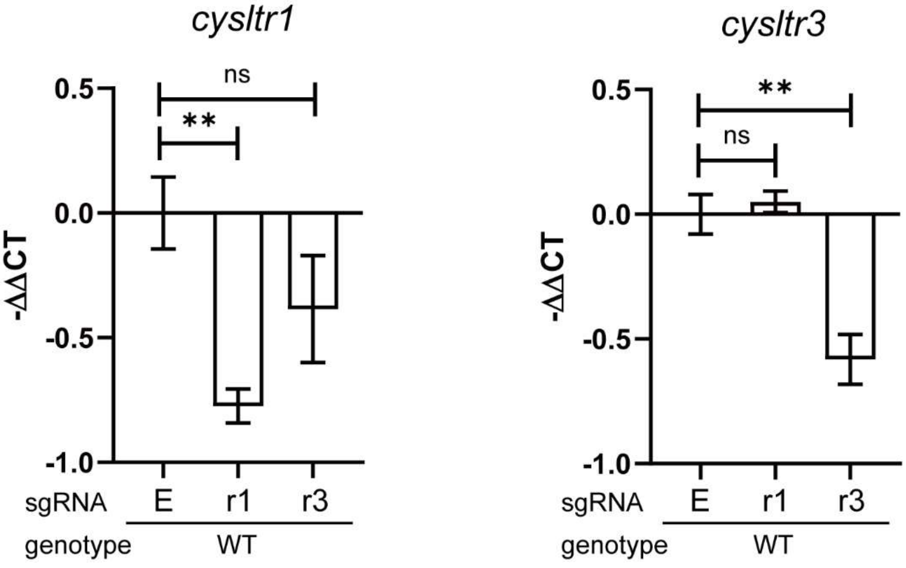

Figure S5. The expression levels of *cysltr1* and *cysltr3* in CRISPRi knockdown embryos were analyzed by quantitative RT-PCR (qRT-PCR). The white bars represent the −ΔΔCt values (normalized by EGFP sgRNA). The y-axis -ΔΔCT is mathematically equivalent to log2(fold change). E, *EGFP* sgRNA; r1, *cysltr1* sgRNA; r3, *cysltr3* sgRNA. ns, no significance; **P < 0.01.

Supplementary Table 1. The oligo sequences used in this study

**Primers**	**Sequence 5'->3'**
**Genotyping**	
Swell1a	GTCACCCAGGACAAGATGATC
	TGGTGCGTGGAAACTTGAACCAG
Swell1b	TTGTGGACACGCAACCTACA
	CACTTGGGGTCGCTTTCCTCTAC
**Cloning**	
Puromycin cassette	AAGATATCGGGTTGCGCCTTTTCCAAGGCAGCCC
	AAAGATCTGGCACCGGGCTTGCGGGTCATGCACC
polyA signal	AAAAGATCTAACTTGTTTATTGCAGCTTATAATG
	AAAAGATCTCAGACATGATAAGATACATTGATGA
Swell1a-Flag	CTCGAGCTCAAGCTTCGATGATTCCCATCACTGA
Swell1b-Flag	CTCGAGCTCAAGCTTCGATGATCCCCATAACTGAG
Flag-reverse	GGGAGAGGGGCGGATCTCACTTGTCGTCATCGTC
**Point mutation**	
twu0421	ACCACCTCAACGTCAGCGTCCCGCTTCA
	GTCTGGTTGACCACCCACTTGCAGGGCA
twu0422	ACAAGAGCTCTCTCTGAAACCGTAGTAG
	GGTGAGTCAAAACACTTGAGTAGGATAG
***in situ* hybridization probe**	
cldn5b	AGAAACTCCTCAAGCACT
	TAATACGACTCACTATAGGAGGTTTAGATGATCAAACC
mrc1a	AAAAATGGCAGAGGGTGT
	TAATACGACTCACTATAGGCAAGATCAGCATCACT
gata1a	ACTGAAGGAGATAAGCAAG
	TAATACGACTCACTATAGGTTCAAATATCACTGA
lyz	GATAAAGCAGATATCAGCAGTG
	TAATACGACTCACTATAGGTACAATTTAGGAAGA
mfap4.1	GATAAACTGAGCTGTTGAGG
	TAATACGACTCACTATAGGAGTTTATTAAAGCTT
myh7	TCTCAAACATGGAACATGTT
	TAATACGACTCACTATAGGCTGATTTAACAGGCTTGTGT
myh6	CCTCAAACATGGAGAGTATT
	TAATACGACTCACTATAGGTTGATTGATGAGGCCTGT
swell1a	AGTTATACGCCACAGGAGCC
	TAATACGACTCACTATAGGCGCTGAAGGTGATGTAGTGC

Supplementary Table 1. The oligo sequences used in this study (continue)

Primers	Sequence 5’->3’
**CRISPR sgRNA**	
cysltr1	ATGTAGTAGAGCACCCGGAGGGG
	GTTACGGAATTCATCGATTGAGG
cysltr3	TGAGAAGCCGACATCCGAAATGG
	GAATACGATGCTGTATGTAATGG
**qPCR**	
cldn5b	GTCATCTCTGCGGTTTTGGG
	TTGGATTTGATAGACTCCGTGTTG
mrc1a	ATGGGTGACTGGATGTGGATT
	AAAAATGTGGCAAGTCCAGCC
myh7	GGAGAGGTCTAATGCGGCTG
	CGGCTTGCGACTCTTCAAAC
myh6	ATTCAAGCTAACCCTGCGCT
	GCTCGTCCCGAAATGAATGC
prox1a	AGCAGAGACTGCGAGCTTTT
	GTCCAGCTTGCAGATGACCT
vegfc	ACCATAAGCTTCGCCAACCA
	ACGTCTCCTCATCCAGCTCT
flt4	CCCTCCAGCACATGTCATTCT
	TGAGTGAAGAACACTGGACGG
klf2a	ACTTCAGCATGTGCGACGAT
	TCATCAGAACGGGCGAACTT
egfl7	TGCAACCAAGCGGTATGTGA
	CTCCACCAGTGTTCCGTGAG
amotl2a	TCGCCAAACTGGTTGAGCA
	GCTCCAGTCTGGTTCTAAGTCG
alox5a	CGGTCTGGGAGACTGTGAAA
	GTTGGTGGGGAGTTTGGGAT
alox5b.1	GGGTCTTTAATGAGGCTAACAGCAG
	GCCTCAGGGAAGCACAGAGA
alox5b.2	CGGACGCTCCAGTTTGCATC
	ACCAGTAGGGCAGCTCCAGA
cysltr1	CCTACCGTCAGACTTCAGCC
	CCGGGAATACGATCGCTAGG

Supplementary Table 1. The oligo sequences used in this study (continue)

**Primers**	**Sequence 5'->3'**
cysltr3	ATGGGACTCACGAGCATGGA
	AAGTCTTCACTTTCCCCGCA
actb1	TTGCTCCTTCCACCATGAAG
	CCTGCTTGCTGATCCACATC
